# Therapeutic Effect of Novel Cyanopyrrolidine-Based Prolyl Oligopeptidase Inhibitors in Rat Models of Amnesia

**DOI:** 10.3389/fchem.2021.780958

**Published:** 2021-12-22

**Authors:** Nikolay N. Zolotov, Igor A. Schepetkin, Tatyana A. Voronina, Vladimir F. Pozdnev, Andrei I. Khlebnikov, Irina V. Krylova, Mark T. Quinn

**Affiliations:** ^1^ Research Zakusov Institute of Pharmacology, Moscow, Russia; ^2^ Department of Microbiology and Cell Biology, Montana State University, Bozeman, MT, United States; ^3^ Institute of Biomedical Chemistry, Moscow, Russia; ^4^ Kizhner Research Center, Tomsk Polytechnic University, Tomsk, Russia; ^5^ Institute of Pharmacy, Altai State Medical University, Barnaul, Russia; ^6^ Zelinsky Institute of Organic Chemistry, Moscow, Russia

**Keywords:** Alzheheimer’s disease, prolyl oligopeptdases, brain, blood brain barrier, cyanopyrrolidine derivatives, antiamnesic activity, conditioned passive avoidance reflex

## Abstract

Prolyl oligopeptidase (POP) is a large cytosolic serine peptidase that is altered in patients with Alzheimer’s disease, Parkinsonian syndrome, muscular dystrophies, and other denervating diseases. Thus, POP may represent a relevant therapeutic target for treatment of neuropsychiatric disorders and neurodegenerative diseases. Here, we report the characterization of five novel cyanopyrrolidine-based compounds (BocTrpPrdN, BocGlyPrdN, CbzMetPrdN, CbzGlnPrdN, and CbzAlaPrdN) and show that they are potent inhibitors of POP and are predicted to penetrate the blood-brain barrier (BBB). Indeed, we show that CbzMetPrdN penetrates the rat BBB and effectively inhibits POP in the brain when administered intraperitoneally. Furthermore, molecular modeling confirmed these compounds likely inhibit POP via interaction with the POP catalytic site. We evaluated protective effects of the cyanopyrrolidine-based POP inhibitors using scopolamine- and maximal electroshock-induced models of amnesia in rats and showed that BocTrpPrdN, BocGlyPrdN, CbzMetPrdN, and CbzGlnPrdN significantly prolonged conditioned passive avoidance reflex (CPAR) retention time when administered intraperitoneally (1 and 2 mg/kg) before evaluation in both models of amnesia, although CbzAlaPrdN was not effective in scopolamine-induced amnesia. Our data support previous reports on the antiamnesic effects of prolinal-based POP inhibitors and indicate an important role of POP in the regulation of learning and memory processes in the CNS.

## Introduction

Proteolytic enzymes play an important role in regulating the function of endogenous peptides. Proteases and peptidases control the concentration of neuropeptides by modulating their formation, modification, and inactivation. Numerous peptidases are present in brain tissue and contribute to the specific functions of neurons or glial cells. Even minor disturbances in the function of these enzymes can contribute to the development of central nervous system (CNS) pathological states, including dementia, Parkinsonism, epilepsy, cerebral ischemia, and Alzheimer’s disease (Candelario-Jalil et al., 2009; Lukasiuk et al., 2011; Tsilibary et al., 2014).

Prolyl oligopeptidase (POP, EC 3.4.21.26) is a large cytosolic serine peptidase that is widely distributed in the CNS, including in the hypothalamus, striatum, hippocampus, cortex, and amygdala. POP specifically cleaves peptide bonds at the carboxylic end of proline residues in proline–containing endogenous peptides [reviewed in (Garcia-Horsman et al., 2007; Mannisto et al., 2007; Peltonen et al., 2011; Nagatsu, 2017)]. For example, POP hydrolyses thyroliberin, vasopressin, neurotensin, substance P, angiotensin, bradykinin, oxytocin, and other regulatory neuropeptides (Garcia-Horsman et al., 2007; Svarcbahs et al., 2019). POP is localized in various cellular compartments, including the perinuclear space, cytoskeletal microtubules, and synaptic contacts and has been reported to play a role in intracellular mechanisms of protein transport and secretion (Morawski et al., 2011). POP is also found in the nuclei of neurons at early stages of development and differentiation, suggesting an as yet unknown function of POP in the regulation of gene expression during neurogenesis (Garcia-Horsman, 2011). POP has been associated with a variety of different neurotransmitters and may be involved in inhibitory and excitatory signal transmission and in thalamocortical and corticothalamic signaling in the brain (Peltonen et al., 2011). This peptidase is also involved in the secretion and degradation of α-synuclein (αSyn), a key neurotoxic protein involved in Parkinson’s disease (Ganguly et al., 2021). For example, Savolainen et al. (2015) reported that POP binds directly to αSyn to enhance its dimerization and thus may serve as a nucleation point for αSyn aggregation. Moreover, POP has been shown to regulate autophagy via protein-protein interaction with protein phosphatase 2A (PP2A) (Svarcbahs et al., 2020). Note that PP2A plays an important role in the pathophysiology of Alzheimer’s disease (Liu et al., 2008; Sontag and Sontag, 2014). POP inhibitors can also prevent aggregation and enhance the clearance of accumulated αSyn (Myöhänen et al., 2012; Dokleja et al., 2014; Savolainen et al., 2014; Savolainen et al., 2015; Kilpelainen et al., 2020). Although POP inhibitors could regulate the effects of POP on autophagy and αSyn aggregation through conformational stabilization of POP, this is not equivalent to inhibiting its proteolytic activity (Kilpelainen et al., 2020).

POP activity correlates with several CNS functions, particularly those that involve memory, learning, and mood related responses (Harwood, 2011; Nazarova et al., 2012; Kumar et al., 2017). On the other hand, POP activity is altered in patients with Alzheimer’s disease, Parkinsonian syndrome, muscular dystrophies, and other denervating diseases (Aoyagi et al., 1990; Garcia-Horsman et al., 2007; Babkova et al., 2017; Svarcbahs et al., 2019). Additionally, brain POP expression has been reported to increase with age (Rampon et al., 2000), although POP enzymatic activity can vary in both directions (Männistö and García-Horsman, 2017). Thus, it is not clear whether POP activity would automatically be elevated under pathological situations.

Data on variations in the activity of proline-specific enzymes in the clinical picture of major depression have served as a basis for the development of a laboratory model of the anxiety–depressive state in rats (Maes et al., 1994; Khlebnikova et al., 2009a; Krupina et al., 2009; Syunyakov et al., 2017). For example, POP activity was increased in several brain structures in this animal model, including the hippocampus, frontal cortex, and striatum (Kushnareva et al., 2011). These observations suggest that manipulation of POP activity and its secondary effects on neuropeptide levels could represent a potential therapeutic approach for treatment of Alzheimer’s disease, memory impairment, and cognitive disorders (Yoshimoto et al., 1987; Kamei et al., 1992; Toide et al., 1995; Portevin et al., 1996; Morain et al., 2002; Jalkanen et al., 2007). In addition, POP located in activated microglia is a potential target for neuroprotection (Natunen et al., 2019). Indeed, previous studies have shown that low molecular weight inhibitors of POP had pronounced neuroprotective, nootropic, antidepressant, and anxiolytic properties (Penttinen et al., 2011; Krupina et al., 2013a). Indeed, three POP inhibitors (JTP-4819, Z-321, and S 17092) are in phase I and/or II clinical trials, thus demonstrating that small molecule POP inhibitors could be safely administered in humans (Svarcbahs et al., 2019).

Alzheimer’s disease is the most prevalent disease of old age leading to dementia. Alzheimer’s disease pathogenesis is complex and involves multiple factors, such as hyperphosphorylated tau, a microtubule assembly protein that is deposited intracellularly in neurofibrillary tangles; β-amyloid plaque formation; and inflammation; ensuing degeneracy; and neuronal cell death (Hannula et al., 2013; Calsolaro and Edison, 2016; Sagar et al., 2019). In early stages, cholinergic neurons are not affected, but with progression of disease, severe loss of acetylcholine has been found to be commensurate with cognitive deficits (Chen and Mobley, 2019), and scopolamine-induced amnesia has been widely used as animal model of Alzheimer’s disease. Scopolamine (competitive muscarinic receptor antagonist) is a tropane alkaloid that inhibits depolarization of muscarinic receptors by acetylcholine and interferes with other neurotransmitter systems (Khurana et al., 2021). This alkaloid also impairs storage and consolidation mechanisms and leads to loss of learning and memory (Gilles and Ertle, 2000).

Most potent and effective POP inhibitors are small molecule nitrile- and prolinal-based compounds (Friedman et al., 1984; Yoshimoto et al., 1987; Jalkanen et al., 2007; Ivanova et al., 2020). Cyanopyrrolidines (nitrile-based inhibitors) have been previously used as cysteine- and serine-reactive electrophilic agents to target cysteine and serine proteases, respectively, where the active-site cysteine or serine undergoes nucleophilic attack on the electrophilic center at the carbon of the nitrile moiety (Pei et al., 2006). Several *N*-acylated glycyl-(2-cyano)pyrrolidines displayed inhibitory activity toward fibroblast activation protein (FAP) (Ryabtsova et al., 2012; Jansen et al., 2013), a serine protease belonging to the prolyl oligopeptidase family (Šimková et al., 2020). CbzMetPrdN, a cyanopyrrolidine-based compound, also inhibited activity of dipeptidyl peptidase IV (DPP-4) (Ivanova et al., 2020).

The catalytic triad of POP consists of Ser554, His680, and Asp641 (Szeltner et al., 2002), and it has been hypothesized that cyanopyrrolidine inhibitors might act through covalent modification of Ser554 in the POP active site (Venalainen et al., 2006). Ser554 can interact with the nitrile group of prolyl nitrile or prolinal residues, forming an imidate or a hemiacetal, respectively and, thus, covalently bind to the inhibitor. KYP-2047 [(2S)-1-[[(2S)-1-(1-oxo-4-phenylbutyl)-2-pyrrolidinyl] carbonyl]-2-pyrrolidinecarbonitrile] and CbzPro-prolinal are the best studied POP inhibitors and exhibit a broad range of biological properties, including neuroprotective, anti-angiogenic, and antitumor effects (Myöhänen et al., 2011; Peltonen et al., 2012; Myöhänen et al., 2017; Natunen et al., 2019; Xu et al., 2019; Scuderi et al., 2021). These inhibitors also exhibited therapeutic effects in animal models of Parkinson’s and Alzheimer’s disease (Yoshimoto et al., 1987; Jalkanen et al., 2007; Svarcbahs et al., 2016). KYP-2047 and JTP-4819 were effective in a model of scopolamine-induced amnesia (Portevin et al., 1996; Toide et al., 1997; Jalkanen et al., 2007; Peltonen et al., 2010), although KYP-2047 improved escape performance (i.e., latency to find the hidden platform and swimming path length) of young, but not older rats in a Morris water maze (Jalkanen et al., 2007).

**FIGURE 1:**
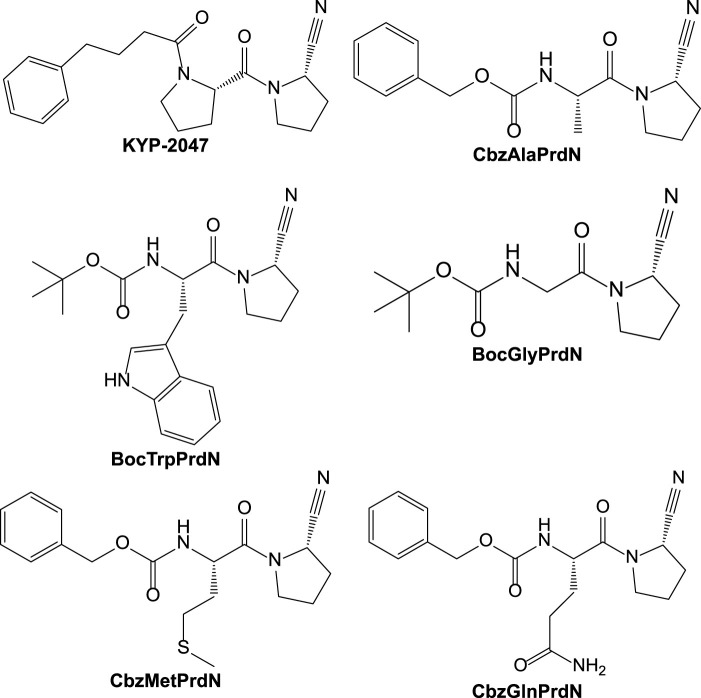
Structures of KYP-2047 and its novel analogs.

In the present study, we synthesized and evaluated the POP-inhibitory activity of five novel KYP-2047 analogs, including BocTrpPrdN, BocGlyPrdN, CbzMetPrdN, CbzGlnPrdN, and CbzAlaPrdN (Figure 1). Molecular modeling suggested these cyanopyrrolidine derivatives bind to POP catalytic site, and we found that CbzMetPrdN penetrates the rat BBB and effectively inhibits POP activity in the brain when administered intraperitoneally. We also evaluated protective effects of these novel cyanopyrrolidine-based POP inhibitors in scopolamine-induced and maximal electroshock (MES)-induced models of amnesia in rats.

## Experimental Section

### Chemistry

Compounds N-benzyloxycarbonyl-L-glutamine (CbzGlnOH) (Yadav et al., 2015), N-(*tert*-butoxycarbonyl)-L-prolinamide (BocProNH_2_) (Wang et al., 2012), N-benzyloxycarbonyl-L-methionyl-L-prolinamide (CbzMetProNH_2_) (Pozdnev, 1995), N-benzoxycarbonyl-L-alanyl-(*2S*)-pyrrolidine-2-carbonitrile (CbzAlaPrdN) (Lawandi et al., 2009), N-(*tert*-butoxycarbonyl)glycyl-(*2S*)-pyrrolidine-2-carbonitrile (BocGlyPrdN) (Jansen et al., 2013), and N-(*tert*-butoxycarbonyl)-L-tryptophanyl-(*2S*)-pyrrolidine-2-carbonitrile (BocTrpPrdN) (Shi, 2017) were synthesized as described in the literature. Reaction progress was monitored by thin layer chromatography with UV detection using silica gel F254 plates (Merck). The synthesized structures were confirmed on the basis of analytical and spectral data. The melting points (m.p.) were determined using an electrothermal Mel-Temp capillary melting point apparatus. Chemical structures of the inhibitors were confirmed by mass spectrometry and NMR; sample purity was >99%.

**N-Benzyloxycarbonyl-L-methionyl-(*2S*)-pyrrolidine-2-carbonitrile (CbzMetPrdN)**. To a solution of 6.5 mmol of CbzMetProNH_2_ in 10 ml of pyridine cooled to 5°C, 1.7 ml (13 mmol) of benzenesulfonyl chloride were added. The reaction mixture was stirred at 15–17°C for 16 h, the solvent was evaporated *in vacuo*, and the residue was dissolved in ethyl acetate. The resulting solution was washed sequentially with H_2_O, 5% H_2_SO_4_, H_2_O, and brine and then dried over MgSO_4_. The solvent was evaporated, and the residue was crystallized from ether to obtain CbzMetPrdN as a white crystalline powder. Yield 78%; m.p. 73°C; specific rotation ([α]_D_) –94.8° (methanol). Found, %: C 60.1; H 6.6; N 11.3. C_18_H_23_N_3_O_3_S. Calculated, %: C 59.83; H 6.42; N 11.62. IR bands, cm^−1^: 3,450 (NH_2_), 1725 (C=O), 1,660 (NH), 2,233 (CN). ^1^H NMR (CDCl_3_) δ ppm: 7.35 (s, 5H), 5.84 (d, 1H), 5.08 (m, 2H), 4.72 (m, 1H), 4.66 (m, 1H), 3.77 (d, 2H), 2.56 (m, 2H), 2.20 (m, 2H), 2.12 (s, 3H), 2.06–1.82 (m, 2H). ESI-MS, m/z: 362.1496 exp. ([C_18_H_23_N_3_O_3_S + H]^+^ = 362.1538 theor.).

**N-Benzyloxycarbonyl-L-glutamine *p*-nitrophenyl ester**. To a solution of 1 mol of CbzGlnOH in dimethylformamide (DMF), 1.1 mol of *p*-nitrophenol and 1.1 mol of dicyclohexylcarbodiimide (DCC) were added at 0°C, stirred for 1 h at 0°C and left for 20 h at room temperature. The precipitated dicyclohexylurea was filtered, washed with ethyl acetate, and the solvent was removed under reduced pressure. The residue was crystallized from ethanol. M.p. 155–156°C [α]_D_ –24° (DMF).

**N-Boc-L-proline nitrile (BocPrdN)**. To a solution of 20 mmol BocProNH_2_ in 20 ml of pyridine at 10°C, 4 ml (31.5 mmol) of benzenesulfonyl chloride were added and stirred for 12 h. The mixture was diluted with H_2_O, extracted with ethyl acetate (100 ml), and the extract was washed sequentially with 0.5 M H_2_SO_4_, H_2_O, and 1 M Na_2_CO_3_ and then dried. The solvent was evaporated, and the residue was crystallized from hexane. Yield 50%, m.p. 47–48°C [α]_D_ –64.0° (methanol).

**L-proline nitrile trifluoroacetate**. CF_3_COOH (2 ml) was added to a solution of 2 g BocPrdN in 2 ml of dichloromethane. After 30 min, the solvent was evaporated, and the residue was crystallized from ether. M.p. 98–99°C. [α]_D_ –19.6° (ethanol), −25.4° (methanol).

**N-Benzyloxycarbonyl-L-glutaminyl-(*2S*)-pyrrolidine-2-carbonitrile (CbzGlnPrdN)**. A solution of proline nitrile trifluoroacetate (0.6 g), 1.2 g of N-Cbz-glutamine *p*-nitrophenyl ester, and 0.5 ml of triethylamine in 5 ml of DMF was stirred for 16 h, diluted with ethyl acetate, washed sequentially with H_2_O, 0.5 M H_2_SO_4_, 1 M Na_2_CO_3_, H_2_O, and brine, and dried with MgSO_4_. The solvent was evaporated, and the residue was recrystallized from chloroform. Yield 30%, m.p. 119–120° [α]_D_ –37.4°. Found, %: C 60.5, H 6.4, N 15.3; C_18_H_22_N_4_O_4_. Calculated, %: C 60.34; H 6.19; N 15.63. IR bands, cm^−1^: 3,450 (NH_2_), 1725 (C=O), 1,660 (NH), 2,233 (CN). ^1^H NMR (CDCl_3_) δ pm: 7.38, 7.30 d (5H); 6.16–6.0 m (1H); 5.58 d (1H); 5.12–5.08 m (2H); 4,72 d, 4.64 d, 4.50 m (2H); 3.86–3.50 m (2H); 2.40–2.0 m (5H); 1.92–1.82 s (2H). ESI-MS, m/z: 359.1714 exp. ([C_18_H_22_N_4_O_4_ + H]+ = 359.1719 theor.).

### *In vivo* Study Solutions

To prepare emulsions of the inhibitors and scopolamine for injection in to animals, the amount of compound powder corresponding to a proper dose for the animal was mixed with 20 μl of Tween 80. Two ml of physiologic NaCl solution was added to create a suspension.

### POP Inhibition Assay

POP was isolated from rat brain, as described previously (Cummins et al., 2011) with modifications. Briefly, rat brain homogenate was precipitated with ammonium sulfate, followed by ion exchange chromatography on DEAE-Sephadex A-50, gel chromatography on Sephadex G-75, and high-performance liquid chromatography on a MonoQ column using FPLC. Based on analysis using sodium dodecyl sulfate polyacrylamide gel electrophoresis (SDS-PAGE), the resulting POP preparation was a homogeneous monomer with molecular weight of 75 kDa. Purified POP was concentrated by ultrafiltration and stored at −80°C.

Enzymatic activity was determined by measuring the release of 7-amino-4-methylcoumarin (AMC) from the synthetic fluorogenic POP substrate Cbz-Ala-Pro-AMC (synthesized at the Institute of Biomedical Chemistry, Moscow, Russia; purity >98%) which is an excellent substrate for POP from rat brain: k_cat_ (sec^−1^) = 161.7 ± 13.5 and K_m_ (μM) = 54 ± 4. The reaction was initiated by addition of the substrate (20 μM) in a final reaction volume of 2 ml. The incubation mixture consisted of 50 μl of the enzyme preparation in 20 mM Tris-HCl buffer (pH 7.5) with 1 mM EDTA, 1 mM dithiothreitol, and 0.1% NP–40; 50 μl of 1 mg/ml substrate solution in DMSO; and 1.9 ml of Tris-HCl buffer. Final concentration of DMSO in the reaction solution was 2.5%, which had no effect on POP enzymatic activity (data not shown). After a 30-min incubation at 37°C, the reaction was stopped by addition of 1 ml of acetate buffer (pH 4.0). The AMC concentration was determined on an LS–5B spectrofluorometer (Perkin–Elmer, United States) with excitation and emission wavelengths of 380 and 460 nm, respectively. For all compounds tested, the concentration of inhibitor that caused 50% inhibition of the enzymatic reaction (IC_50_) was calculated by plotting % inhibition versus logarithm of inhibitor concentration (at least 5-6 points).

### Animals

All manipulations with experimental animals were performed in accordance with international and Russian regulatory documents: Order of the Ministry of Health of the Russian Federation No. 199n of April 1, 2016 and Directive 2010/63/EU of the European Parliament and the Council of the European Union for the Protection of Animals Used in Scientific purposes. Experiments were performed on 3 month old male Wistar rats. Animals were housed in accordance with the sanitary and epidemiological rules of SR 2.2.1.3218–14 “Sanitary and epidemiological requirements for the design, equipment and maintenance of experimental biological clinics (vivariums).” The experiments were approved by the Commission on Biomedical Ethics of Zakusov Institute of Pharmacology (Protocol #1 of January 20, 2017). Rats were housed in groups of five animals per cage (57 × 36 × 20 cm) in standard laboratory conditions (ambient temperature of 22 ± 2°C, relative humidity of 60%, and 12:12 h light-dark cycle) in cages with sawdust bedding, standard rodent feed, and ad libitum water access.

### Measurement of the Tissue Concentration of CbzMetPrdN and POP Activity in the Brain

The total drug concentrations for CbzMetPrdN and POP activity in the brain were determined as a function of time at 0, 20, 40, 60, 80, and 120 min (*n* = 3 for each time-point) after a single 20 mg/kg intraperitoneal (*i.p.*) administration of CbzMetPrdN. The test animals were sacrificed, and their brains were harvested and immediately homogenized in phosphate buffer saline.

For measurement of the brain tissue concentration of CbzMetPrdN, the homogenate was mixed with an equal volume of methanol and centrifuged at 3,000 g for 10 min. The supernatant was concentrated to 2 ml, followed by solid phase extraction in a Retain AX SPE cartridge (Thermo Fisher Scientific). The cartridge was washed with 1 ml of 5% ammonia solution and then with 1 ml of methanol. The sample was eluted with 1 ml of 2% trifluoroacetic acid in methanol and analyzed using a Gilson 41 HPLC system (Gilson, United Kingdom) with a 125 × 4 mm Nucleosil C18 column (Macherey-Nagel). The column was washed for 3 min with 4 mM hexanesulfonate in water (pH 3.5), followed by a 0–100% gradient of 4 mM hexanesulfonate in acetonitrile:water (50:50) % (v/v) over 10 min. The concentration of the POP inhibitor is expressed in ng of CbzMetPrdN per mg of brain protein.

POP activity in the brain tissue homogenate was measured using Cbz-Ala-Pro-AMC, as described above, and is presented as nmole AMC released/min/mg of brain protein. Protein concentrations of the supernatants were determined with a Bio-Rad protein assay kit.

### Conditioned Passive Avoidance Reflex (CPAR)

The development of CPAR was performed on a standard passive avoidance device from Lafayette Instrument Co. (United States). The device consists of a small platform located at a distance of 1 m from the floor, illuminated by a special lamp, and a dark chamber with an electrode floor. During learning, a rat was placed on the small platform in front of the entrance to the dark chamber. Immediately after entering the dark chamber, the rat received a painful stimulation from an electric current of 0.6 mA through the electrode floor lasting 10 s or until the rat ran out of the chamber (learning). A rat was considered trained if it did not enter the dark chamber for the next 30 s. If the rat entered the dark chamber, the training was repeated. A test for the refiex reproduction was performed after 24 h. In this case, the rat was placed on a lighted area, and step-through latency time (STL) of its entry into the dark chamber was recorded. The observation time was 3 min. The criterion for refiex reproduction was considered to be the STL of entry into the dark box.

### Models of Amnesia and Experimental Protocol

Experiments were performed using two rat models of amnesia. In the first model, effects of POP inhibitors on scopolamine-induced amnesia were studied. The series included eighteen groups of animals (137 rats). Rats in the negative control group (group 1, *n* = 8) received 2 ml of vehicle (physiological saline solution containing 1% Tween 80) *i.p.* 40 min before the CPAR learning. Rats in the non-treated group (group 2, *n* = 7) received scopolamine (1 mg/kg, *i.p.* in 2 ml of vehicle) 20 min before the CPAR learning. Rats of groups 3–17 received a single injection of POP inhibitor at doses of 0.5, 1, and 2 mg/kg, *i.p*. (three groups of animals for each inhibitor), including CbzAlaPrdN (groups 3–5, *n* = 8 in each), BocTrpPrdN (groups 6–8, *n* = 8 in each), BocGlyPrdN (groups 9–11, *n* = 8 in each), CbzMetPrdN (groups 12–14, *n* = 6 in each), and CbzGlnPrdN (groups 15–17, *n* = 8 in each) followed (after 20 min) by administration of scopolamine (1 mg/kg in 2 ml of vehicle, *i.p*.) 20 min before the CPAR learning. Rats in group 18 (*n* = 8) received piracetam (300 mg/kg) followed (after 20 min) by administration of a single injection of scopolamine (1 mg/kg in 2 ml of vehicle) 20 min before the CPAR learning.

In the second model, effects of POP inhibitors on MES-induced amnesia were studied. The series included fifteen groups of animals (110 rats). Rats in the negative control group (group 1, *n* = 8) received 2 ml of vehicle (physiological saline solution containing 1% Tween 80) *i.p.* 30 min before the CPAR learning. Rats in the positive control group (group 2, *n* = 7) received MES just after CPAR learning. MES was induced using an electric shock generator from Harvard Apparatus (Holliston, MA, United States) consisting of a current (250 V, 120–122 20 mA, delivered for 0.1 s) by way of corneal electrodes. Rats of groups 3–14 received a single injection of POP inhibitor at doses of 0.5, 1, and 2 mg/kg, including BocTrpPrdN (groups 3–5, *n* = 7 in each), BocGlyPrdN (groups 6–8, *n* = 6 in each), CbzMetPrdN (groups 9–11, *n* = 8 in each), and CbzGlnPrdN (groups 12–14, *n* = 8 in each) 40 min prior to the CPAR learning (three groups of animals for each inhibitor) with following MES just after CPAR learning. Rats in group 15 (*n* = 8) received piracetam (300 mg/kg, *i.p*.) 50 min before the CPAR learning and MES just after CPAR learning.

### Statistical Analysis

Statistical analysis was performed using Statistica 10.0. The normality of the distribution was evaluated using the Shapiro-Wilk criterion with subsequent assessment of the homogeneity of variances according to the Leven criterion. In the case of a normal distribution in the experimental groups and homogeneity of variances between the groups, further processing was performed using the method of parametric statistics with Student’s *t* test. Values were considered statistically significant when *P* was <0.05.

### Molecular Modeling

Compound structures were created using ChemOffice 2016 software pre-optimized with the MM2 force field and saved in Tripos MOL2 format. The ligand structures were then imported into the Molegro Virtual Docker 6.0 program (MVD). The structure of POP complexed with (*6S*)-1-chloro-3-[(4-fluorobenzyl)oxy]-6-(pyrrolidin-1-ylcarbonyl)pyrrolo[1,2-*a*]pyrazin-4(6*H*)-one (Haffner et al., 2008) was downloaded from the Protein Data Bank (PDB code 3DDU) and also imported into MVD. Co-crystallized water molecules were removed from the 3DDU structure during importing. A search space for docking was defined in the POP binding site as a sphere of radius 12 Å positioned at the geometric center of gravity of the ligand. The investigated compounds were docked into the binding site. MolDock score functions were applied with a 0.3 Å grid resolution. Ligand flexibility was accounted for with respect to torsion angles auto-detected in MVD. Structure of the protein was considered rigid. The “Internal HBond” and “sp^2^-sp^2^ torsions” options were activated in the “Ligand evaluation” menu of the MVD Docking Wizard. Three hundred docking runs were performed for each molecule. The option “Return multiple poses for each run” was enabled, and the post-processing options “Energy minimization” and “Optimize H-bonds” were applied after docking. Similar poses were clustered at a RMSD threshold of 1 Å.

The physicochemical properties of selected compounds were computed using SwissADME (http://www.swissadme.ch) (Daina et al., 2017).

Molecular modeling for the POP inhibitors was used for calculation of the following parameters: aLogP was calculated using HyperChem 7 software based on additive scheme (Ghose and Crippen, 1987), tPSA (topological polar surface area) was calculated according to atomic increments (Ertl et al., 2000), the number of rotatable bonds N_rot_ was determined from the structural formulae. The predicted ability of the compounds to penetrate the blood-brain barrier (BBB) was determined in accordance with the model proposed by Suenderhauf et al. (2012).

## Results and Discussion

### Chemistry

**SCHEME 1:**
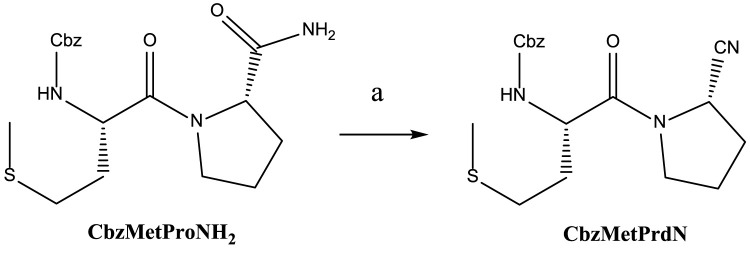
Synthesis of CbzMetPrdN. Reagents and conditions: a) PhSO_2_Cl, in pyridine, 15–17°C, 16 h.

We synthesized two novel compounds: CbzMetPrdN and CbzGlnPrdN. The other proline nitrile derivatives (CbzAlaPrdN, BocGlyPrdN, and BocTrpPrdN) were synthesized as previously reported (Lawandi et al., 2009; Jansen et al., 2013; Shi, 2017); however, this is the first report of their effects on POP activity. CbzMetPrdN was synthesized as shown in Scheme 1 from CbzMetProNH_2_. This amide was converted into the target nitrile by the action of benzenesulfonyl chloride in pyridine. This final stage is a modification of the methodology by Cobb et al. (2005), who used TsCl instead of PhSO_2_Cl to obtain N-protected proline nitriles.

**SCHEME 2:**
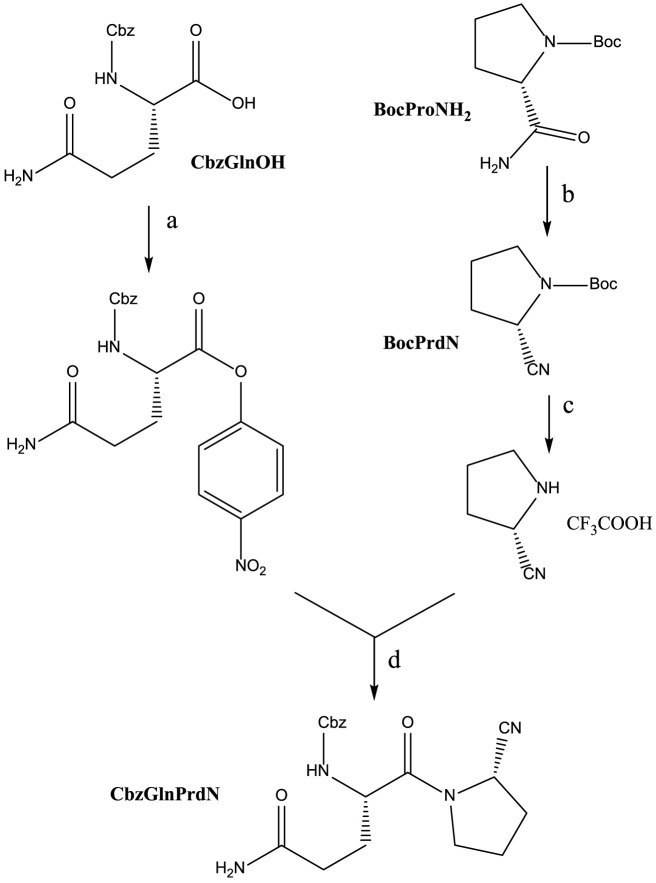
Synthesis of CbzGlnPrdN. Reagents and conditions: a) *p*-C_6_H_6_NO_2_, DCC, in DMF, 0°C, 1 h; b) PhSO_2_Cl, in pyridine, 10°C, 12 h; c) CF_3_COOH, 30 min; d) Et_3_N, in DMF, 16 h.

CbzGlnOH and BocProNH_2_ were used as starting materials for the synthesis of CbzGlnPrdN according to Scheme 2. The conversion of Boc-protected prolylamide into the corresponding nitrile was performed again by addition of benzenesulfonyl chloride (see synthesis of CbzMetPrdN described above). Subsequent deprotection of the nitrile gave L-proline nitrile trifluoroacetate, which was further involved in the reaction with *p*-nitrophelyl ether of Cbz-protected glutamine.

### POP Inhibitory Activity of the Cyanopyrrolidine Derivatives

Five cyanopyrrolidine-based derivatives were evaluated for POP inhibitory activity in comparison with the known POP inhibitor KYP-2047, and the results are reported in Table 1. All cyanopyrrolidine-based compounds were potent POP inhibitors, with inhibitory activity in the low nanomolar range and the most active compounds being CbzMetPrdN and CbzGlnPrdN (IC_50_ ∼ 2 nM). Inhibitory activity of the reference POP inhibitor KYP-2047 (Tocris Bioscience, San Diego, CA, United States) was in same concentration range as reported previously by Van der Veken et al. (2012) (IC_50_ = 6 ± 4 nM) and was similar to the activity of most of our new cyanopyrrolidine-based POP inhibitors.

**TABLE 1:** POP inhibitory activity of novel cyanopyrrolidine derivatives and KYP-2047.

Compound	IC_50_ (nM)
CbzAlaPrdN	5.2 ± 0.3
BocTrpPrdN	9.0 ± 0.9
BocGlyPrdN	12.0 ± 3
CbzMetPrdN	2.1 ± 0.6
CbzGlnPrdN	2.0 ± 0.3
KYP-2047	2.9 ± 0.5

### Molecular Docking of the POP Inhibitors Into POP

**FIGURE 2:**
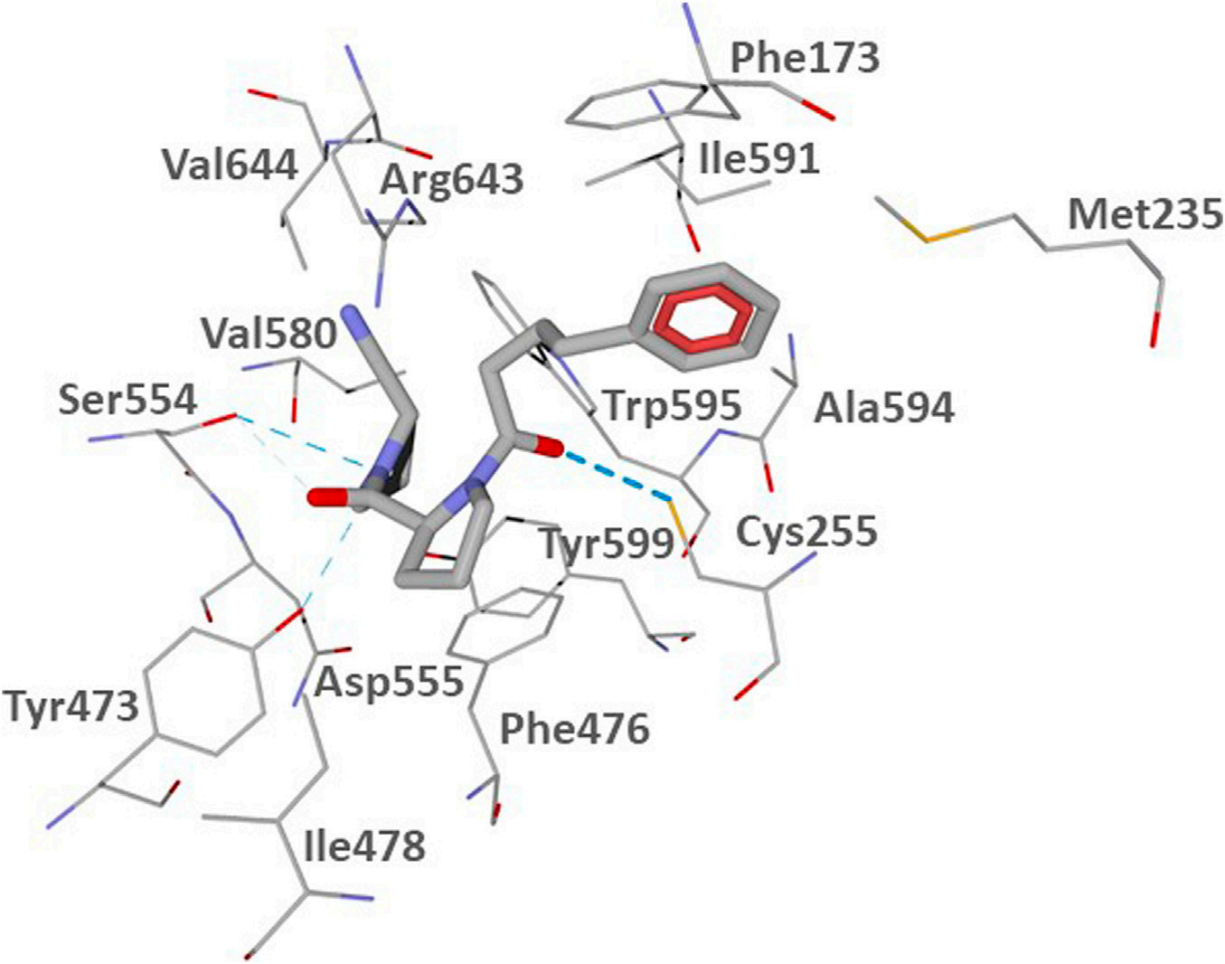
Docking pose of inhibitor KYP-2047 into the POP binding site (PDB code 3DDU). Residues within 3 Å of the pose are visible. H-bonds are shown as blue dashed lines.

The POP binding site contains a catalytic triad of serine protease consisting of Ser554, His680, and Asp641. Ser554 can interact with the prolyl nitrile group, forming an imidate and, thus, covalently binding the inhibitor (Szeltner et al., 2002; Venalainen et al., 2006). To assess potential of the investigated compounds to interact with the POP binding site in a manner favorable for covalent interaction of their nitrile groups with Ser554, we performed molecular docking studies of CbzGlnPrdN and CbzMetPrdN. For comparative purposes, we also docked the reported POP inhibitor KYP-2047 (Kilpelainen et al., 2019). We used the 3DDU structure from the Protein Data Bank which contains POP inhibitor (*6S*)-1-chloro-3-[(4-fluorobenzyl)oxy]-6-(pyrrolidin-1-ylcarbonyl)pyrrolo[1,2-*a*]pyrazin-4(6*H*)-one complexed in the POP binding site. A pyrrolidine ring of the co-crystallized inhibitor is located in the vicinity of Ser554, and the molecule itself occupies key cavities of the site denoted as S1, S2, and S3 (Guardiola et al., 2018). As reported by Kilpelainen et al. (2019), a similar location was obtained for the docking pose of KYP-2047, which occupies cavities S1-S3 and has the prolyl nitrile group directed towards Ser554, providing an opportunity for a covalent bond formation via nucleophilic attack of the serine oxygen center onto the nitrile carbon atom. In our work, we attempted to use Molegro Virtual Docker (MVD) software to reproduce the docking pose of KYP-2047 previously obtained with the Glide program (Kilpelainen et al., 2019). The pose obtained by us with MVD is shown in Figure 2.

According to our results (Figure 2), the nitrile-substituted pyrrolidine ring of KYP-2047 is located near Ser554 in pocket S1. Analogously to the previous report (Kilpelainen et al., 2019), the obtained pose also enters pocket S2 between Phe476 and Arg643. The phenyl group is protruded to pocket S3 in the vicinity of Phe173 and Cys255 (compare our Figure 2 with Kilpelainen et al., 2019). Pocket S3 is also flanked by other hydrophobic residues Met235, Ile591, and Ala594 (Guardiola et al., 2018). The distance from the hydroxyl oxygen atom of Ser544 of the catalytic triad to the nitrile carbon atom of the docked inhibitor KYP-2047 is 3.311 Å. Such a proximity of the two reaction centers ensures the potential of covalent bonding between the ligand and POP.

**FIGURE 3:**
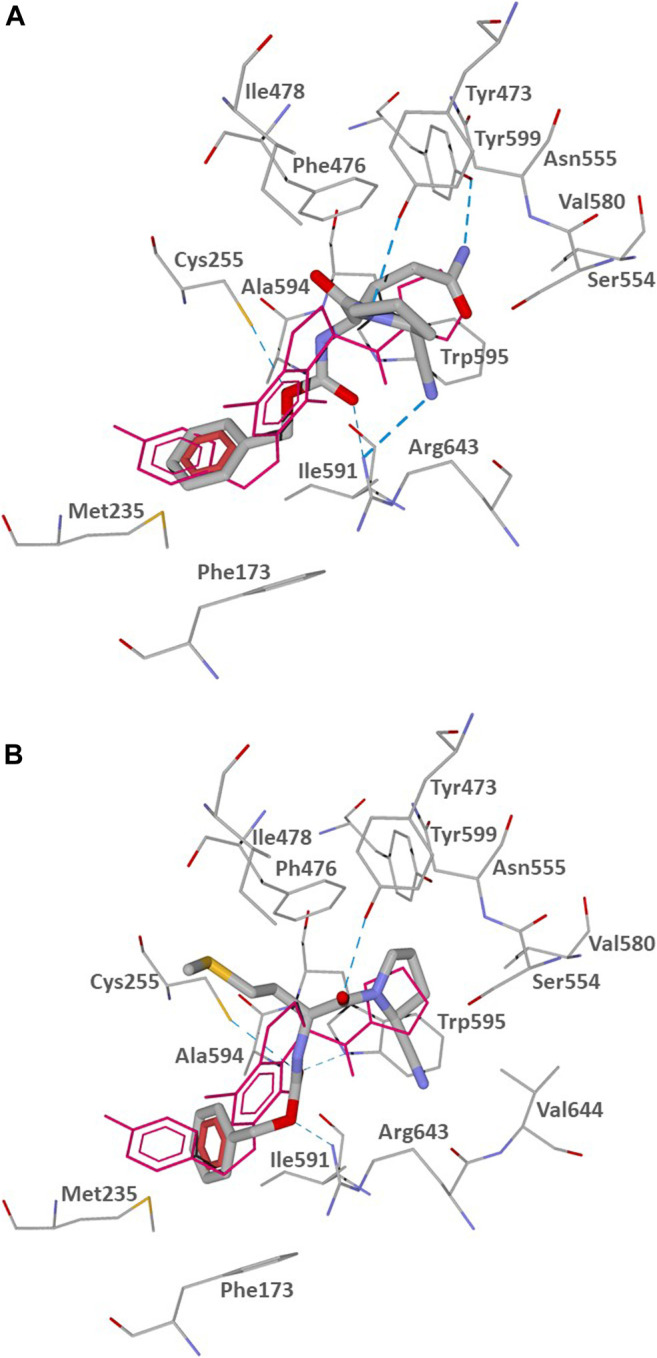
Docking poses of CbzGlnPrdN **(A)** and CbzMetPrdN **(B)** into the POP binding site (PDB code 3DDU). Residues within 3 Å of the pose are visible. H-bonds are shown as blue dashed lines. The co-crystallized POP inhibitor from the 3DDU structure is depicted in thin magenta lines.

The docking poses of CbzGlnPrdN and CbzMetPrdN are shown in Figure 3. Both CbzGlnPrdN and CbzMetPrdN are oriented within the POP-binding site similarly to KYP-2047 and the co-crystallized inhibitor from the 3DDU structure. Thus, nitrile-substituted proline moieties of the inhibitors fall into pocket S1 close to Ser544. Central parts of the ligand backbones and terminal Cbz phenyl groups occupy pockets S2 and S3, respectively (Figure 3).

The docking results indicate that CbzGlnPrdN is H-bonded to Arg643 *via* the Cbz carbonyl group and nitrile nitrogen atom. Hydrogen bonds are also formed with the proline nitrogen and glutamine amino groups to Tyr473 and Tyr599, respectively. The oxygen atom of the Cbz protecting group H-bonded is weakly H-bonded to Cys255 (Figure 3A). CbzMetPrdN is anchored by hydrogen bonds to Cys255 and Trp595 via the carbonyl oxygen atom of the Cbz group. The other oxygen of this group is H-bonded to Arg643, while the proline oxygen atom forms a hydrogen bond with Tyr473 (Figure 3B).

In the docking poses obtained, the distances between the nitrile carbon and the hydroxyl oxygen atom of Ser554 are 3.348 and 3.400 Å for compounds CbzGlnPrdN and CbzMetPrdN, respectively. These values are favorable for nucleophilic attack of Ser554 onto the nitrile group. Hence, the mechanism of POP inhibition by the investigated compounds likely involves covalent bonding to the enzyme with the formation of imidates similar to prolyl nitrile derivatives (Venalainen et al., 2006; Kilpelainen et al., 2019).

### BBB Permeability for the Derivatives and POP Inhibition in the Brain by CbzMetPrdN

Three molecular descriptors are used for prediction BBB permeability, including aLogP, topological polar surface area (tPSA), and the number of rotatable bonds (N_rot_) (Table 2).

**TABLE 2:** Predicted properties of POP inhibitors.

Property	CbzAlaPrdN	BocTrpPrdN	BocGlyPrdN	CbzMetPrdN	CbzGlnPrdN	KYP-2047
Formula	C_16_H_19_N_3_O_3_	C_21_H_26_N_4_O_3_	C_12_H_19_N_3_O_3_	C_18_H_23_N_3_O_3_S	C_18_H_22_N_4_O_4_	C_20_H_25_N_3_O_2_
M.W.	301.34	382.46	253.30	361.46	358.39	339.43
Heavy atoms	22	28	18	25	26	25
Fraction Csp^3^	0.44	0.48	0.75	0.50	0.44	0.55
Rotatable bonds	7	8	6	10	10	7
H-bond acceptors	4	4	4	4	5	3
H-bond donors	1	2	1	1	2	0
MR	83.79	110.11	68.96	101.00	96.31	103.44
tPSA	82.43	98.22	82.43	107.73	125.52	64.41
iLogP	2.48	2.92	2.11	2.71	2.06	2.94
aLogP	1.85	2.36	0.37	1.83	0.62	2.31
BBB permeation	Yes	Yes	Yes	Yes	Yes	Yes

Abbreviations: M.W., molecular weight (g/mol); MR, molar refractivity; tPSA, topological polar surface area (Å2); iLogP, lipophilicity obtained using physics-based method described by Daina et al. (2014); aLogP, lipophilicity evaluated by increment-based method developed by Ghose and Crippen (1987) and calculated using HyperChem seven software; BBB, blood–brain barrier.

In accordance with the classification model (Suenderhauf et al., 2012), these values allowed us to determine that KYP-2047 and all five novel POP inhibitors represent compounds that would be predicted to penetrate through the BBB. Indeed, it was found previously that KYP-2047 penetrates mouse and rat brains (Jalkanen et al., 2011a; Jalkanen et al., 2014).

**FIGURE 4:**
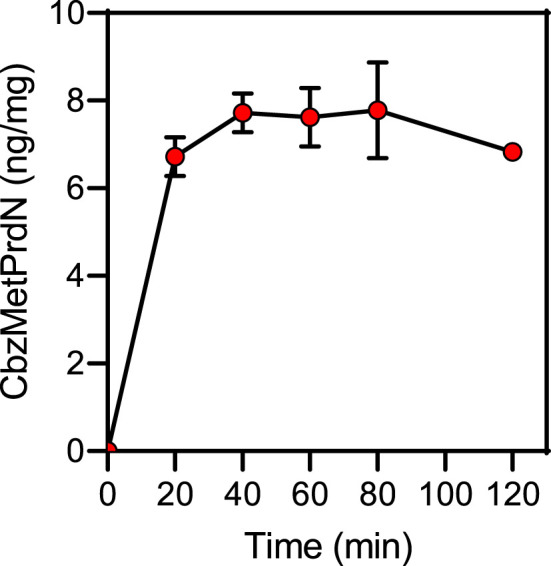
Time-dependent accumulation of CbzMetPrdN in brain tissue after a single *i.p.,* administration (20 mg/kg) of the compound (*n* = 3 for each time point).

We also directly evaluated the concentration-time profile of CbzMetPrdN accumulation in rat brain tissue and found that this compound accumulated in the brain after a single *i.p.* administration (20 mg/kg) (Figure 4).

**FIGURE 5:**
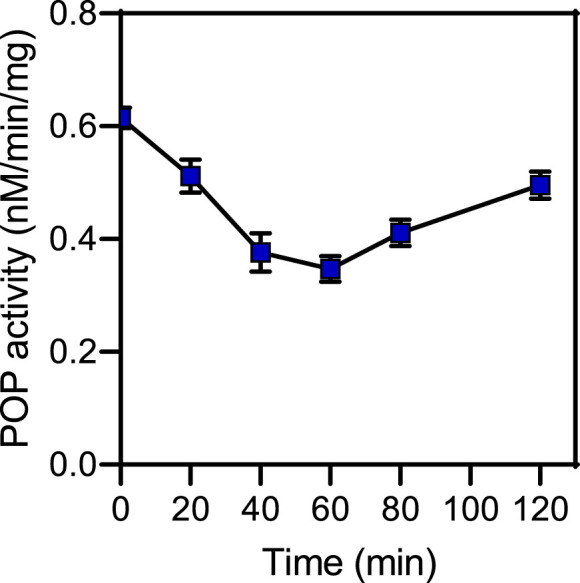
POP activity in brain tissue after a single *i.p*., administration (20 mg/kg) of CbzMetPrdN (*n* = 3 for each time point).

Rat brain POP activity as a function of time after CbzMetPrdN administration is shown in Figure 5. The maximal inhibition was observed at 60 min after administration of the inhibitor, which is the time point with maximal concentration of CbzMetPrdN in the brain.

### Antiamnesic Activity of Cyanopyrrolidine-Based POP Inhibitors

**FIGURE 6:**
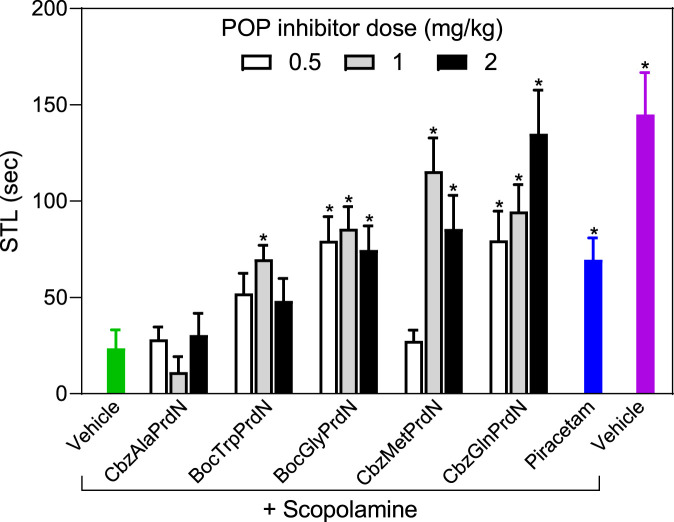
Effect of novel cyanopyrrolidine-based POP inhibitors on scopolamine-induced amnesia of conditioned passive avoidance reflex (CPAR). Rats in the group without treatment received scopolamine (1 mg/kg) 20 min before CPAR learning (green bar). Rats in the treatment groups received the indicated POP inhibitor (at doses 0.5, 1, or 2 mg/kg; white, gray, and black bars) or piracetam (300 mg/kg; blue bar) followed by a single injection of scopolamine (1 mg/kg) 20 min prior to CPAR learning. Rats in the negative control group received vehicle 40 min before CPAR learning (magenta bar). * Significant difference (*p* < 0.05) with nontreated rats (scopolamine only).

The antiamnesic properties of prolinal-based POP inhibitors and KYP-2047 have been demonstrated previously (Yoshimoto et al., 1987; Jalkanen et al., 2007). Here we tested the antiamnesic activity of our novel cyanopyrrolidine-based POP inhibitors using models of scopolamine and MES-induced amnesia (Ader et al., 1972; Jalkanen et al., 2007; Lavrov et al., 2020). Rats treated with scopolamine showed a decrease in the latency period (23.5 ± 9.6 s) when compared with vehicle-treated rats (145.0 ± 21.7 s). We found that administration of BocGlyPrdN, CbzMetPrdN, and CbzGlnPrdN significantly prolonged CPAR retention time when administered at doses 1 and 2 mg/kg before evaluation in the scopolamine-induced model of amnesia, although CbzAlaPrdN was not effective at all doses. A clear tendency toward antiamnesic activity was also observed for BocTrpPrdN. However, due to the significant scatter of measures for individual animals, the differences did not reach significance at doses 0.5 and 2 mg/kg. Importantly, these POP inhibitors were more active than the standard nootropic piracetam (300 mg/kg) (Figure 6).

**FIGURE 7:**
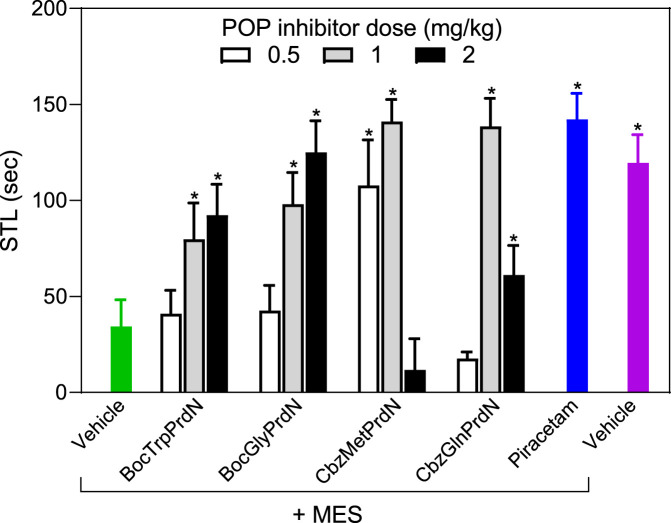
Effect of novel cyanopyrrolidine-based POP inhibitors on MES-induced amnesia of conditioned passive avoidance reflex (CPAR). Rats in the group without treatment received MES just after learning (green bar). Rats in the treatment groups received the indicated POP inhibitor (0.5, 1, or 2 mg/kg; white, gray, and black bars) or piracetam (300 mg/kg; blue bar) 40 min before CPAR learning, followed by MES just after learning. MES was induced using an electric shock generator (250 V, 120–122 20 mA, delivered for 0.1 s) by way of corneal electrodes. Rats in the negative control group received vehicle (magenta bar). * Significant difference (*p* < 0.05) with nontreated animals (MES only).

The four POP inhibitors that showed activity in scopolamine-induced amnesia (BocTrpPrdN, BocGlyPrdN, CbzMetPrdN, and CbzGlnPrdN) were evaluated for antiamnesic activity in MES-induced amnesia. MES resulted in a decrease in STL time (34.3 ± 14.0 s) in comparison with rats from the control group (119.6 ± 14.7 s). At a dose of 1 mg/kg, CbzMetPrdN, and CbzGlnPrdN had potent antiamnesic activity, which was comparable with the antiamnesic activity of piracetam. Although BocTrpPrdN and BocGlyPrdN were less potent in comparison with piracetam (300 mg/kg), these POP inhibitors exhibited antiamnesic activity at doses of 1 and 2 mg/kg (Figure 7).

POP is a serine protease that has been studied particularly in the context of neurodegenerative diseases, including Alzheimer’s disease (Mannisto et al., 2007). Over the past decade, many drugs have been evaluated for targeting POP in the treatment of Alzheimer’s disease. Among of them, several POP inhibitors with different scaffolds were effective in a model of scopolamine-induced amnesia (Li et al., 1996; Portevin et al., 1996; Toide et al., 1997; Jalkanen et al., 2007). Herein, we demonstrated potent POP inhibitory activity of novel cyanopyrrolidine-based derivatives, with the most active being CbzMetPrdN and CbzGlnPrdN. We also estimated the BBB permeability of these POP inhibitors and found that they were predicted to penetrate the BBB, which is supported by our results showing that CbzMetPrdN penetrates the rat BBB and effectively inhibits POP in the brain when administered intraperitoneally.

We found that administration of POP inhibitors, including BocTrpPrdN, BocGlyPrdN, CbzMetPrdN, and CbzGlnPrdN, significantly improved scopolamine- and MES-induced memory impairments in the CPAR model, with the most potent being CbzMetPrdN. It should be noted that CbzMetPrdN was previously studied in a preclinical model of Parkinsonian syndrome, where it exhibited neuroprotective activity and abolished depressive symptoms caused by the neurotoxin 1-methyl-4-phenyl-1,2,3,6-tetrahydropyridine (MPTP) (Khlebnikova et al., 2009b; Krupina et al., 2009; Khlebnikova et al., 2012; Khlebnikova et al., 2013a; Khlebnikova et al., 2013b; Kalinina et al., 2019; Krupina et al., 2013b). Thus, CbzMetPrdN would be an ideal compound to evaluate in other models of CNS disease.

Our results show that novel cyanopyrrolidine-based derivatives can inhibit POP enzymatic activity *in vitro* and *in vivo* and that administration of these compounds can be beneficial in rat models of amnesia. However, the exact mechanisms involved are still not fully elucidated. For example, Jalkanen et al. (2011b) proposed that POP is not responsible for *in vivo* cleavage of substance P or neurotensin and that positive cognitive effects associated with POP inhibitors are not mediated through elevated extracellular levels of these peptides. In addition, POP can participate in protein-protein interactions with various neuronal proteins (aSyn, α-tubulin, and growth-associated protein-43) independently of peptidase activity (Schulz et al., 2005; Di Daniel et al., 2009; Szeltner et al., 2010; Lambeir, 2011; Savolainen et al., 2015). Thus, POP seems to play multiple roles in brain physiological functions. In any case, our studies clearly show that POP inhibitors have beneficial antiamnesic effects, suggesting that they may represent novel and potential therapeutic agents for treating dementia in Alzheimer’s disease.

## Data Availability

The raw data supporting the conclusion of this article will be made available by the authors, without undue reservation.
